# Characteristics of the heme catabolic pathway in mild unconjugated hyperbilirubinemia and their associations with inflammation and disease prevention

**DOI:** 10.1038/s41598-017-00933-y

**Published:** 2017-04-07

**Authors:** Christine Mölzer, Marlies Wallner, Carina Kern, Anela Tosevska, René Zadnikar, Daniel Doberer, Rodrig Marculescu, Karl-Heinz Wagner

**Affiliations:** 1 University of Aberdeen, School of Medicine, Medical Sciences and Nutrition, Institute of Medical Sciences, Foresterhill, Ashgrove Road West, AB25 2ZD Aberdeen UK; 2 University of Applied Sciences, FH JOANNEUM, Institute of Dietetics and Nutrition, Alte Poststraße, 149, 8020 Graz Austria; 3 University of Vienna, Faculty of Life Sciences, Department of Nutritional Sciences, Althanstraße 14 (UZA2), 1090 Vienna, Austria; 4 Medical University of Vienna, Center for Physiology and Pharmacology, Institute of Pharmacology, Währingerstraße 13A, 1090 Vienna, Austria; 5 Central Institute of Medical and Chemical Laboratory Diagnostics, University Clinics Innsbruck, 6020 Innsbruck, Austria; 6 Medical University of Vienna, Department of Clinical Pharmacology, Vienna General Hospital, Währinger Gürtel 18–20, 1090 Vienna, Austria; 7 Medical University of Vienna, Clinical Institute of Laboratory Medicine, Vienna General Hospital, Währinger Gürtel 18–20, 1090 Vienna, Austria; 8 Department of Molecular, Cell and Developmental Biology, UCLA, 610 Charles E. Young Drive East, Los Angeles, CA 90095 USA

## Abstract

Heme catabolism exerts physiological functions that impact health through depressing inflammation. Upon reactive pathway progression, as in Gilbert’s Syndrome (GS; UGT1A1*28 polymorphism), aggravated health effects have been determined. Based on lower inflammation and improved metabolic health reported for GS, inter-group differences in heme catabolism were explored. Therefore, a case-control study including 120 fasted, healthy, age- and gender matched subjects with/without GS, was conducted. Genetic expressions of *HMOX-1* and *BLVRA* were measured. Additionally participants were genotyped for those polymorphisms that are known (UGT1A1*28) or likely (*HMOX-1* microsatellites) to impact bilirubinemia. Intracellular interleukins (IL-6, IL-1β, TNFα), circulatory C-reactive protein (CRP), serum amyloid A (SAA) and haptoglobin (Hpt) were analysed as inflammatory markers. To assess intracellular heme oxygenase 1 (HO-1) isolated PBMCs were used. In GS vs. C, inflammation markers were significantly decreased. This was supported by an altered heme catabolism, indirectly reflecting in elevated unconjugated bilirubin (UCB; main phenotypic feature of GS) and iron, decreased hemopexin (Hpx) and Hpt and in up-regulated biliverdin reductase (*BLVRA*) gene expressions. Moreover, *HMOX* (GT)_n_ short alleles were non-significantly more prominent in female GS individuals. Herewith, we propose a concept to elucidate why GS individuals encounter lower inflammation, and are thus less prone to oxidative-stress mediated diseases.

## Introduction

Chronic, oxidative stress mediated (inflammatory) diseases including the conditions of diabetes mellitus type 2 (DM 2), cardiovascular diseases (CVD) and cancer these days represent a major burden to both health and economy. These and other chronic (metabolic) disorders are influenced to a large extent by aspects of lifestyle, including diet and bodily activity. Albeit, there exists a 3–12% section of the population^1^ that, in addition to the known benefits arising from maintaining a healthy lifestyle, genetically appears to be less prone to such health threats. Predicated on this group’s inherited particularity in the *UGT1A1* promoter, commonly referred to as Gilbert’s Syndrome (GS; Morbus Meulengracht; UGT1A1*28 polymorphism), carriers of this mutation statistically encounter a lower prevalence of inflammatory diseases, leading to a later mortality thereof^2^. As among populations of Caucasian ancestry, the *28 allele is most commonly found^3^, specifically this mutation was screened for in the present study. It is however possible, that secondary to this target additional underlying *UGT1A1* polymorphisms had been present (e.g. UGT1A1*6; G > A; G71R missense mutation; most commonly found in Asian populations^3, 4^). So far over one hundred *UGT1A1* genetic variants have been described^5^, and there are reports that the *28 allele by itself is indeed necessary but not sufficient to phenotypically lead to GS^6–8^, but rather acts in combinations with other *UGT1A1* mutations.

Mechanistic studies focusing on hyperbilirubinemic carriers of the *28 allele (GS) have recently reported longevity on a cellular level (through longer telomeres based on less telomeric attrition^9^), as well as a boosted energy metabolism (through activation of the *AMPK* pathway^10^) as possible molecular explanations.

A plethora of reports have furthermore brought the heme catabolic pathway into play to causally explain this confirmed phenomenon of disease prevention^11^ and improved metabolic health in GS individuals^12^. This physiological degradation process takes place in the reticulo-endothelial system of mammals, ultimately resulting in the production of unconjugated bilirubin (UCB). The latter is specifically abundant in GS individuals, representing the only measurable biochemical characteristic of GS, and therefore an important diagnostic marker of the condition.

When heme is enzymatically degraded, involving the pathway’s rate-limiting inducible enzyme heme oxygenase 1 (HO-1; heat-shock protein family 32^13^), as well as biliverdin reductase (BLVRA), UCB is produced in addition to three other separate signalling molecules^14^. These intermediates with antioxidant (biliverdin IXα; carbon monoxide, CO) and anti-microbial (ferrous iron, Fe^2+^) properties have been suggested to impact and improve inflammation^12^, and act *via* distinct molecular targets to influence cell function^14^.

While there is general agreement that these by-products of heme catalysis collaborate with UCB, the latter has been repeatedly demonstrated as the leading mediator of HO-1 antioxidant functions^15^. UCB is known for its antioxidant, anti‐inflammatory and anti‐complement effects *in vitro* ^16, 17^ and *in vivo* ^11, 18, 19^, all of which influence secondary affections of the body. In this respect HO-1's cross-talk with UCB has been a major focus in the development of potential therapeutic strategies to reverse the clinical complications of obesity, since the enzyme’s repression has been negatively associated with inflammatory conditions^20, 21^. Upon cytokine release stress response genes, including *HMOX-1*, are rapidly induced. The absence of intracellular HO-1 therefore leads to chronic systemic inflammation, emphasizing the necessity of this enzyme in immune response^22^.

Rapid turnover of heme through its enzymatic degradation furthermore bears the advantage of removing its availability to pathogens^23^ and of protecting tissues from pro-oxidant heme attacks^24, 25^. Thus, HO-1 is considered the only enzyme that degrades pro-oxidant heme to yield antioxidant products^20^. A well equilibrated heme balance is of specific importance as a critical regulator of molecular pattern recognition and cellular redox status^26^. Tied to and secondary to heme, tissue-protection is further implemented through the action of hemopexin (Hpx) and haptoglobin (Hpt), two high-affinity binding proteins to free heme and to hemoglobin respectively. A reactive decrease in both markers indicates substantial heme catalysis and a drop in Hpt is considered the most sensitive indicator of hemolysis^27, 28^. A reactively boosted heme turnover (induced by its catabolites) has been suggested to occur in the condition of GS, and is likely based on UCB-induced mild hemolysis^12, 29^, ultimately reflecting in decreasing levels of Hpt.

To further explore (GS-specific) characteristics of the heme catalytic pathway, in the present study not only a range of specific biochemical markers, but also microsomal enzyme levels together with their respective underlying genetic expressions (*HMOX-1*, *BLVRA*) were measured. Furthermore the deeper genetic background of heme catabolism was investigated, through *HMOX* (GT)_n_ genotyping (GT-repeats). Findings were linked to the condition of GS, to better explain how genetic patterns could cross-talk *in vivo* on a molecular level. *HMOX-1* (GT)_n_ microsatellite polymorphisms vary from 12–40 repeats^30^, with fragment lengths of 23–30 being the most common^31^. It is generally believed that shorter (GT)_n_ repeats, as compared to longer ones, have higher transcriptional activity and thus account for higher HO-1 protein levels^20^.

The potential relevance of GT fragment lengths ((GT)_n_) and their implications for HO-1 functioning have previously been experimentally linked to inflammation and related diseases. Class L ((GT)_n_ = 30) alleles have been associated with susceptibility to oxidative stress mediated diseases, compared to short fragment lengths^32, 33^. In a human trial by Endler *et al*., carriers of the short allele ((GT)_n_ = 22) had higher UCB as well as higher HDL levels^34^, making these individuals less susceptible to oxidative stress mediated diseases. Similarly, in a study of Yamada *et al*., short (<25 GT) repeats (in contrast to longer ones) have been associated with an increased HO-1 up-regulation in response to inflammatory stimuli^32^.

To this end, an observational case control study including 120 healthy age- and gender-matched male and female subjects with and without GS was conducted. The study’s main aim was to further explore the known phenomenon of a lower inflammatory status in relation to specific genetic patterns (UGT1A1*28- and *HMOX* genotypes) that are inherent to or in likely crosstalk with the condition of GS. Therefore a molecular approach was used focusing on the genetic basis of the heme degradation pathway, together with the analysis of a range of specific biochemical markers and particularly the inflammatory parameters of intracellular IL-6, IL-1β, TNFα, and circulatory CRP, SAA and Hpt/Hpx.

## Results

### Description of the BiliHealth study population and comparison between GS- and C subjects

Subjects between study groups did not significantly differ in terms of age distribution. These and a range of related descriptive findings have been published before by our group^9, 10^ (Supplementary Table S1).

As for body composition, a lower BMI was found in both male and female GS subjects, relative to C (p 0.023 and 0.017). Female GS subjects furthermore presented with a significantly lower body fat percentage than C (p 0.011).

As expected and crucial in terms of the study design, significant inter-group differences were found for UCB (p 0.000) and the respective distribution of the number of TA-repeats (i.e. UGT1A1*28 polymorphism; p 0.000). These genotype distributions reflect what has been reported in the literature^6, 35^.

By trend, heme levels were lower in male GS individuals versus C (p 0.088). This result was not retained in female subjects, where heme levels did not differ between the groups.

Blood pressure was similar between the study groups. No significant differences between GS and C were found in both of the gender groups.

The same applies to unspecific liver parameters (AST, ALT, GGT, LDH) which were found to be equal between GS and C individuals in both of the gender groups. This result importantly confirms the absence of any underlying inflammatory hepatic condition.

### Comparison of parameters of the heme catabolic pathway between GS- and C subjects

#### All subjects

As mentioned, no significant differences were found in heme concentrations (Supplementary Table S1). The same applies to *BLVRA* and the *HMOX* gene expressions, *HMOX*-genotype, HO-1 protein concentrations and the number of GT-repeats (Table 1).

**Table 1:** Parameters of the heme catabolic pathway, immunology and hematology, including all (male and female) subjects from the BiliHealth study.

Male & Female	Variable	Mean° (±sd)/median^^^ (IQR)		*p-value*
		*GS*	*C*	
***Heme catabolic parameters***	*HMOX* expr. [RQ]^	0.97 (0.33) n = 53	0.94 (0.32) n = 51	*0.301*
	*HMOX* genotype/GT-repeats [bp]^	27.5 (3.5) n = 56	29.0 (3.5) n = 55	*0.471*
	HO-1/2 BL [rfU]°	196 (±72) n = 60	207 (±73) n = 60	*0.398*
	HO-1/2 induced (BR) [x-fold]^	0.34 (0.50) n = 59	0.36 (0.50) n = 59	*0.840*
	HO-1/2 induced (H) [x-fold]^	0.10 (0.30) n = 59	0.07 (0.2) n = 59	*0.834*
	*BLVRA* expr. [RQ]^	0.83 (0.25) n = 54	0.74 (0.32) n = 54	*0.173*
***Immunological and hematological parameters***	IL-6 BL [rfU]^	2.4 (0.4) n = 57	2.6 (0.4) n = 59	0.001*
	IL-1β BL [rfU]^	1.3 (0.4) n = 57	1.5 (0.3) n = 59	0.011*
	TNFα BL [rfU]°	25.7 (±5.5) n = 58	25.8 (±5.1) n = 59	*0.919*
	IL-6 induced [x-fold]°	8 (±4) n = 57	8 (±4) n = 59	*0.887*
	IL-1β induced [x-fold]^	21 (15) n = 56	19 (11) n = 59	*0.509*
	TNFα induced [x-fold]°	7 (10) n = 57	8 (10) n = 58	*0.290*
	CRP [mg/dL]^	0.05 (0.06) n = 57	0.07 (0.07) n = 54	0.039*
	Hemopexin [mg/dL]°	84 (±10) n = 51	88 (±14) n = 57	*0.109*
	Haptoglobin [mg/dL]^	76 (46) n = 51	96 (65) n = 57	0.013*
	Plasma iron [µg/dL]^	162 (69) n = 58	121 (56) n = 57	0.000*
	Ferritin [µg/L]^	91 (135) n = 58	105 (112) n = 60	*0.576*
	Hematocrit [%]^	42 (6) n = 59	41 (4) n = 60	*0.386*
	Hemoglobin [g/dL]°	14.7 (±1.3) n = 59	14.4 (±1.3) n = 59	*0.278*
	Reticulocytes abs. [G/L]°	50.7 (±14.8) n = 58	48.1 (±16.4) n = 57	*0.380*
	COHb [%]^	1.20 (0.30) n = 60	1.15 (0.30) n = 60	*0.892*
	SAA [mg/L]^	3.9 (0.0) n = 58	3.9 (0.7) n = 57	0.042*
	Uric acid [mg/dL]°	5.4 (±1.1) n = 60	5.2 (±1.2) n = 60	*0.447*

Values are specified as applies according to distribution of data. For parametric variables, means° ± sd are shown, for non-parametric data, medians^^^ (50^th^ percentiles) and inter-quartile range (IQR) are displayed. Comparison of means for parametric data or of ranks (for non-parametric data) was completed using independent samples t-test or Mann-Whitney-U-test. *p-value: significant on a 5% level of significance; ^T^p-value: trend on a 10% level of trend. Abbreviations: GS: Gilbert’s syndrome; C: Controls; *HMOX* expr. [RQ]: Heme oxygenase gene expression as relative quantification [RQ to cDNA pool]; [bp] base pairs; HO-1/2 [rfU]: intracellular heme oxygenase in PBMCs [relative fluorescence]; HO-1/2 induced (BR)/(H): fold-increase in intracellular heme oxygenase, induced by incubation with unconjugated bilirubin (BR) or heme (H) overnight, relative to baseline; *BLVRA* expr.: Biliverdin reductase gene expression as relative quantification [RQ to cDNA pool]; IL-6 BL/IL-1β BL/TNFα BL [rfU]: baseline (BL) intracellular (PBMCs) levels of interleukins; IL-6 induced/IL-1β induced/TNFα induced [x-fold]: fold-increase in intracellular (PBMCs) levels of interleukins upon LPS-stimulation, relative to baseline; CRP: C-reactive protein; Reticulocytes abs. (G/L): absolute reticulocyte count (10^9^ cells/L; Giga/L); COHb: Carbonyl hemoglobin; SAA: Serum amyloid A.

#### Male subjects

In male GS individuals *BLVRA* gene expression was significantly higher than in C (p 0.038). The number of GT-repeats did not significantly differ between GS and C (p 0.955) (Supplementary Table S2).

#### Female subjects

With respect to markers of the heme catabolic pathway in female subjects, none of the aforementioned inter-group differences were retained. Also for GT-repeats there were no significant inter-group differences (p 0.276) (Supplementary Table S3).

### Comparison of immunological and hematological parameters between GS- and C subjects

#### All subjects

**Figure 1:**
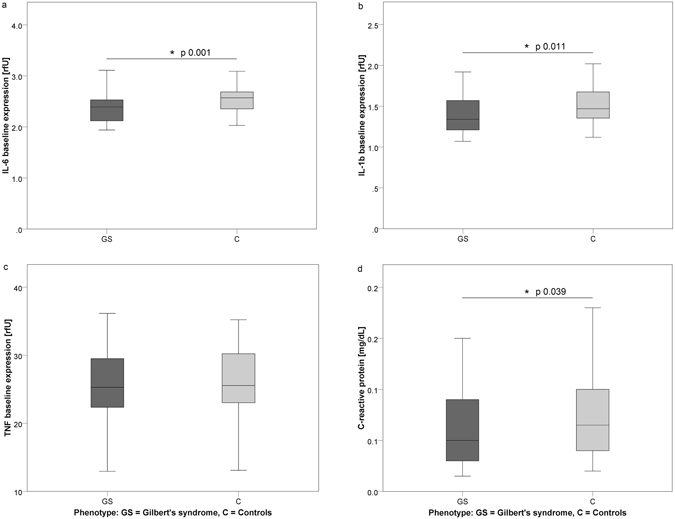
Differences in the inflammatory status between GS and C individuals (all subjects). Figure 1 (**a**–**d**) illustrates inter-group differences in the inflammatory parameters of intracellular IL-6, IL-1β, TNFα and circulating CRP (Phenotype: GS: dark grey, C: light grey). Abbreviations: IL-6/IL-1β: intracellular interleukins 6/1β; TNFα: intracellular tumor necrosis factor; CRP: circulatory C-reactive protein; GS: Gilbert’s syndrome; C: control subjects. *Indicates significant difference on a 5% level of error.

For both genders, significant inter-group differences were found in terms of the intracellular interleukins, IL-6 and IL-1β. Levels were significantly lower in GS individuals as compared to C (p 0.001 and 0.011). The same result applied to levels of circulatory CRP (p 0.039), SAA (p 0.042) and haptoglobin (Hpt; p 0.013). Measures of the inflammatory status are depicted in Fig. 1 and Supplementary Figure S1. Hemopexin (Hpx) was relatively lower in GS individuals, however, this result did not reach significance (p 0.109). Plasma iron was significantly elevated in the GS group (p 0.000) (Table 1).

#### Male subjects

The lower baseline inflammatory status previously reported for the entire GS study population was retained in male subjects and reflected in lower levels of intracellular IL-6 and IL-1β in GS individuals (p 0.000 and 0.002). Also, plasma iron levels were higher in male GS than in C (p 0.000). The same inter-group relation was true by trend for levels of hematocrit (p 0.070) (Supplementary Table S2).

#### Female subjects

In female GS individuals, circulatory CRP (p 0.019), SAA (p 0.009), Hpt (p 0.001) and intracellular TNFα inducibility upon LPS stimulation (p 0.044) were significantly lower than in C. Furthermore, levels of Hpx were relatively lower in female GS individuals. However, this result did not reach statistical significance (p 0.196). As had been reported above for male individuals, levels of plasma iron were also significantly higher in female GS versus C subjects (p 0.025) (Supplementary Table S3).

### *HMOX-1* (GT)_n_-repeats (GT fragment length)

**Figure 2:**
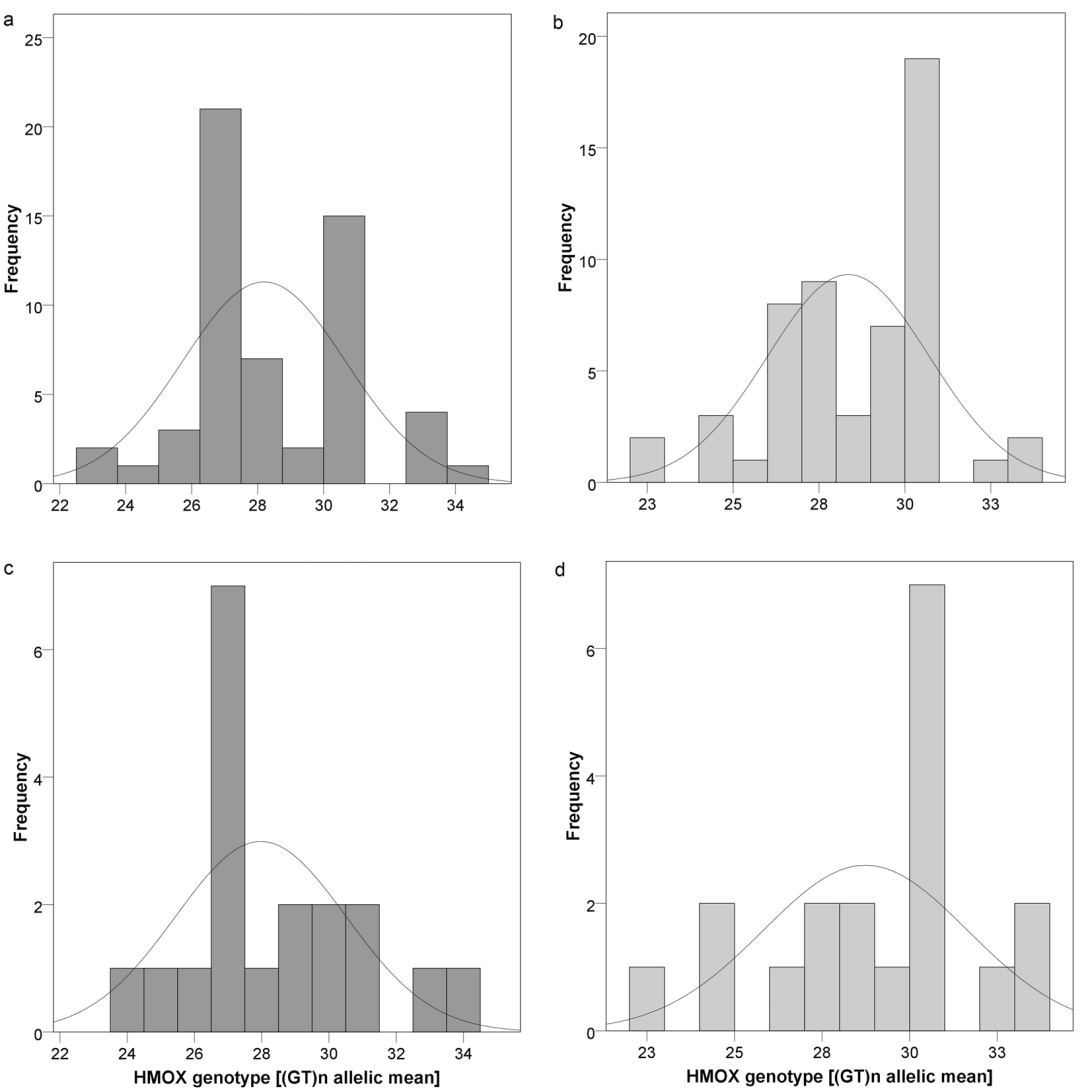
(**a**–**d**) Histograms showing frequency of *HMOX*-genotypes (classified as SS, SM, SL, MM, ML) stated in the BiliHealth study population. Figure 2 shows statistical distribution and frequency of respective *HMOX*-genotypes, expressed as (GT)_n_ allelic mean, between the study groups. (**a** and **b**) Male and female (all) subjects (Phenotype: GS: dark grey; C: light grey). (**c** and **d**) Female subjects only (Phenotype: GS: dark grey; C: light grey). Data distribution proposes a non-significant higher abundance of SM alleles in GS individuals, whereas the MM variant appeared to be more prominent in C subjects. This non-significant observation suggests a (gender-specific) higher frequency of shorter GT-repeats in GS individuals, which would be worth pursuing in a larger study collective. Classification of GT-fragment lengths (S < 26, L > 30) was performed following Chang *et al*.^72^.

*HMOX-1* microsatellites were analysed in terms of GT-fragment lengths, their relative abundance and distribution throughout the study population at hand. The results revealed presence of five genotypic classes within the two study groups (GS, C), namely SS (short/short), SM (short/medium), SL (short/long), MM (medium/medium) and ML (medium/long) alleles. Distributions of the respective genotypes can be found in Table 2. No statistical significance was reached concerning inter-group differences in mean GT-fragment lengths (Table 1). However, non-significant observations suggest a more frequent occurrence of the SM genotype in GS individuals, with the MM variant being more prominent in C subjects. The SM genotype was present in nearly 50% of female GS subjects, whereas roughly the same share of female C carried the MM genotype (Fig. 2). This observation proposes a non-significant higher frequency of shorter GT-repeats in GS. No such distribution pattern was found in males.

**Table 2:** Distribution of *HMOX* genotypes (GT fragment length) between the study groups (GS, C).

*All subjects* (*n* = *111*; *100*%)	(GT)_n_-repeats [S < 26; L > 30 bp]°	Distribution of *HMOX* genotypes and corresponding UCB levels			
		GS n (%)	GS UCB [µM]	C n (%)	C UCB [µM]
	SS	3 (5.4)	37.8 (±13.3)	3 (5.5)	8.1 (±2.9)
	SM	24 (42.9)*	33.4 (±8.3)	19 (34.5)	8.6 (±3.4)
	SL	3 (5.4)	37.2 (±6.4)	2 (3.6)	8.8 (±0.4)
	MM	19 (33.9)	31.7 (±10.5)	25 (45.5)*	9.9 (±3.7)
	ML	7 (12.5)	32.7 (±10.7)	6 (10.9)	8.5 (±3.4)
***Males*** **(** ***n*** ** = ** ***73*** **;** ***100*** **%)**					
	SS	2 (5.4)	41.5 (±16.5)	2 (5.6)	9.3 (±2.9)
	SM	15 (40.5)*	35.8 (±9.3)	15 (41.7)*	8.9 (±2.9)
	SL	2 (5.4)	40.4 (±4.2)	1 (2.8)	9.1 (±0.0)
	MM	15 (40.5)*	32.4 (±11.3)	15 (41.7)*	10.5 (±3.9)
	ML	3 (8.1)	37.5 (±8.0)	3 (8.3)	8.0 (±4.2)
***Females*** **(** ***n*** ** = ** ***38*** **;** ***100*** **%)**					
	SS	1 (5.3)	30.6 (±0.0)	1 (5.3)	5.6 (±0.0)
	SM	9 (47.4)*	29.5 (±4.4)	4 (21.1)	7.1 (±3.2)
	SL	1 (5.3)	30.6 (±0.0)	1 (5.3)	8.5 (±0.0)
	MM	4 (21.1)	28.9 (±7.4)	10 (52.6)*	9.2 (±3.3)
	ML	4 (21.1)	29.1 (±12.7)	3 (15.8)	8.9 (±3.1)

Table 2 specifies the distribution of *HMOX*-genotypes in terms of GT fragment lengths (GT-repeats) in the respective study groups (all subjects and divided by gender), and the corresponding UCB levels (mean ± sd). No statistical significance was reached. However, non-significantly in GS subjects shorter alleles (SM) occurred more frequently than in C, where the MM genotype was more common. The effect was only present in females. UCB levels non-significantly decreased, as GT-repeats increased. °Cut-off levels for classification of GT fragment lengths were adopted from Chang *et al*.^72^. Abbreviations: SS: short/short; SM: short/medium; SL: short/long; MM: medium/medium; ML: medium/long GT fragment length (repeats); GS: Gilbert’s Syndrome; C: Controls. *Most frequently occurring genotype in respective group.

### Inter-variable connection of inflammatory parameters and heme degradation, is merely established *via* UCB and the genetic expressions of the upstream regulators *HMOX* and *BLVRA*

**Figure 3:**
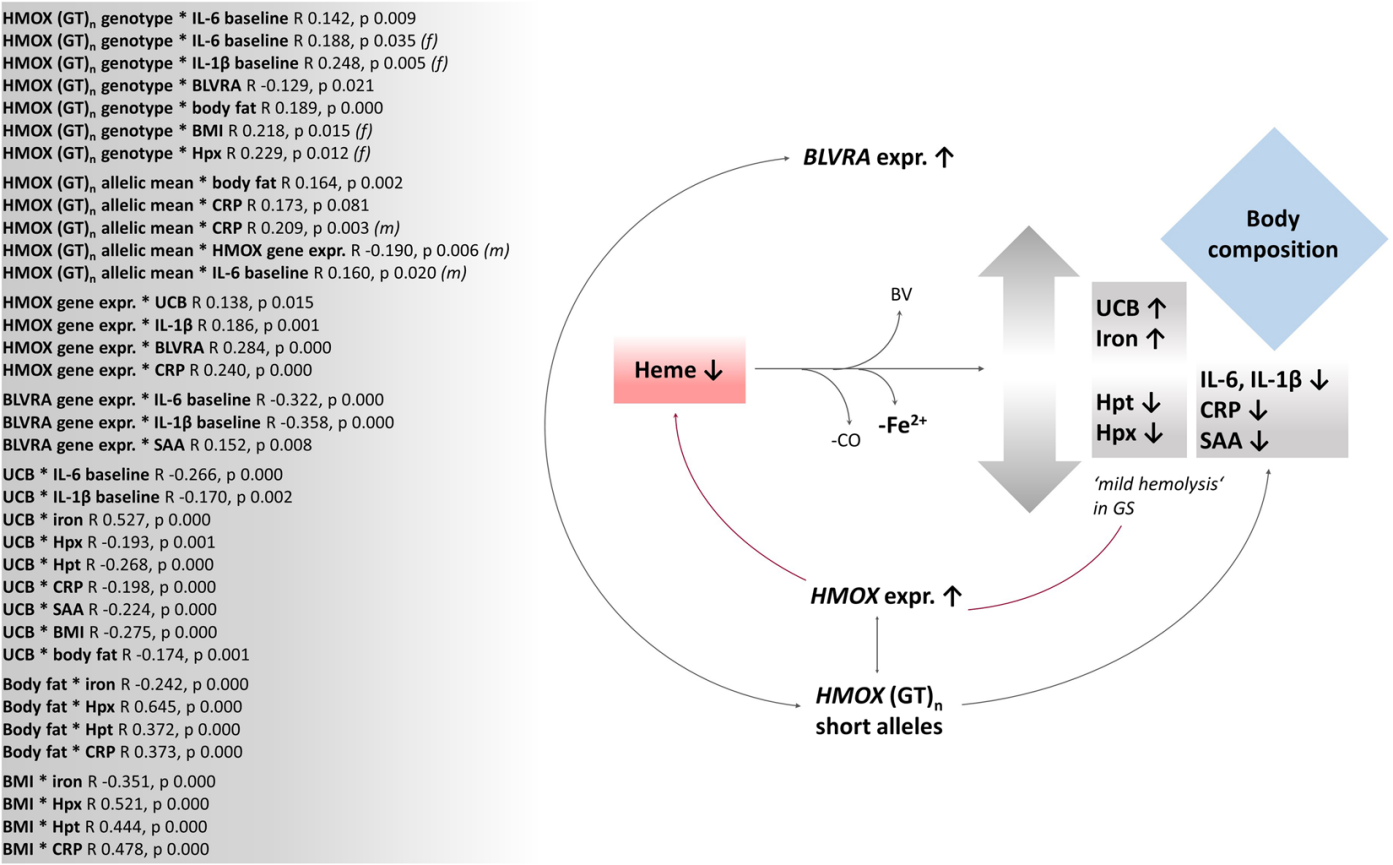
Graphical summary of inter-variable connections for the entire BiliHealth study population. Figure 3 illustrates statistical connections of variables of interest. Bivariate correlations were calculated for the entire study population using the model of Spearman’s rho. R coefficients and p-values (p ≤ 0.05) are presented in the box and are summarised in a graphical model. (*f*): effect valid for female subjects only; (*m*): effect valid for male subjects only. Abbreviations: UCB: unconjugated bilirubin; *UGT1A1*: *UGT1A1* genotype; IL-6/IL-1β: interleukins 6 and 1β; *BLVRA* expr.: genetic expression of biliverdin reductase; *HMOX* expr.: genetic expression of heme oxygenase; *HMOX* (GT)_n_: heme oxygenase GT fragment lengths expressed as genotype (short, medium, long) or as mean fragment length of the two alleles; Hpt: haptoglobin; Hpx: hemopexin; CRP: C-reactive protein; SAA: serum amyloid A; BV: biliverdin IXα; CO: carbon monoxide; Fe^2+^: ferrous iron; BMI: body mass index.

For reasons of statistical validity and power, the entire dataset was analysed for inter-variable connections. A graphical summary of the correlations found is presented in Fig. 3, generating an idea as to how those analysed parameters could be networking on a physiological level. A detailed list of significant inter-variable associations, expressed as correlation coefficients (R) with corresponding p-values (in brackets), is provided in the figure (box) and subsequently described.

To start with UCB as the crucial determinant of the study design, and main phenotypic feature of GS, negative associations with the inflammatory markers of IL-6, IL1β, CRP, SAA as well as with markers of body composition (BMI and BF) were found. Both of which were negatively correlated to the general inflammatory parameter of CRP, in addition to Hpx and Hpt which are the two most sensitive markers of mild hemolysis. An illustration of this can be found in Supplementary Figure S1. In confirmation of a postulated theory of mild UCB-mediated hemolysis in GS^12, 36, 37^ UCB has as well been found negatively correlated to Hpx and Hpt. Furthermore, a connection between Hpx and the *HMOX* genotype was found in female GS, where short GT-repeats were non-significantly more frequent than in C subjects.

Interesting and meaningful correlations were found between BF/BMI and iron, and most markedly between UCB and iron. These findings emphasize an altered heme catabolic pathway in GS, essentially involving the action of the enzyme BLVRA. Gene expression of the latter was found to be in crosstalk with *HMOX* gene expression, as well as in connection with its underlying genetics *via* a negative relation between *BLVRA* gene expression and *HMOX* genotype. Besides its significant positive relation to UCB, the *HMOX* gene expression was furthermore negatively associated with the mean GT fragment length, emphasizing the non-significant observation of shorter GT-repeats in (female) GS individuals.

All three variables (*HMOX* gene expression, *HMOX* (GT)_n_ allelic mean, *HMOX* (GT)_n_ genotype) were connected to inflammatory markers such as interleukins, CRP, as well as to parameters of body composition. On a physiological level this emphasizes the crosstalk between the main product of heme degradation (UCB) and inflammation, possibly *via* impacting body composition and secondarily metabolic health.

Finally, a similar and related association was found between *BLVRA* expression and inflammatory markers (IL-6, IL-1β and SAA).

### UCB as well as *HMOX* and *BLVRA* genetic expressions have explanatory power for markers of inflammation

**Figure 4:**
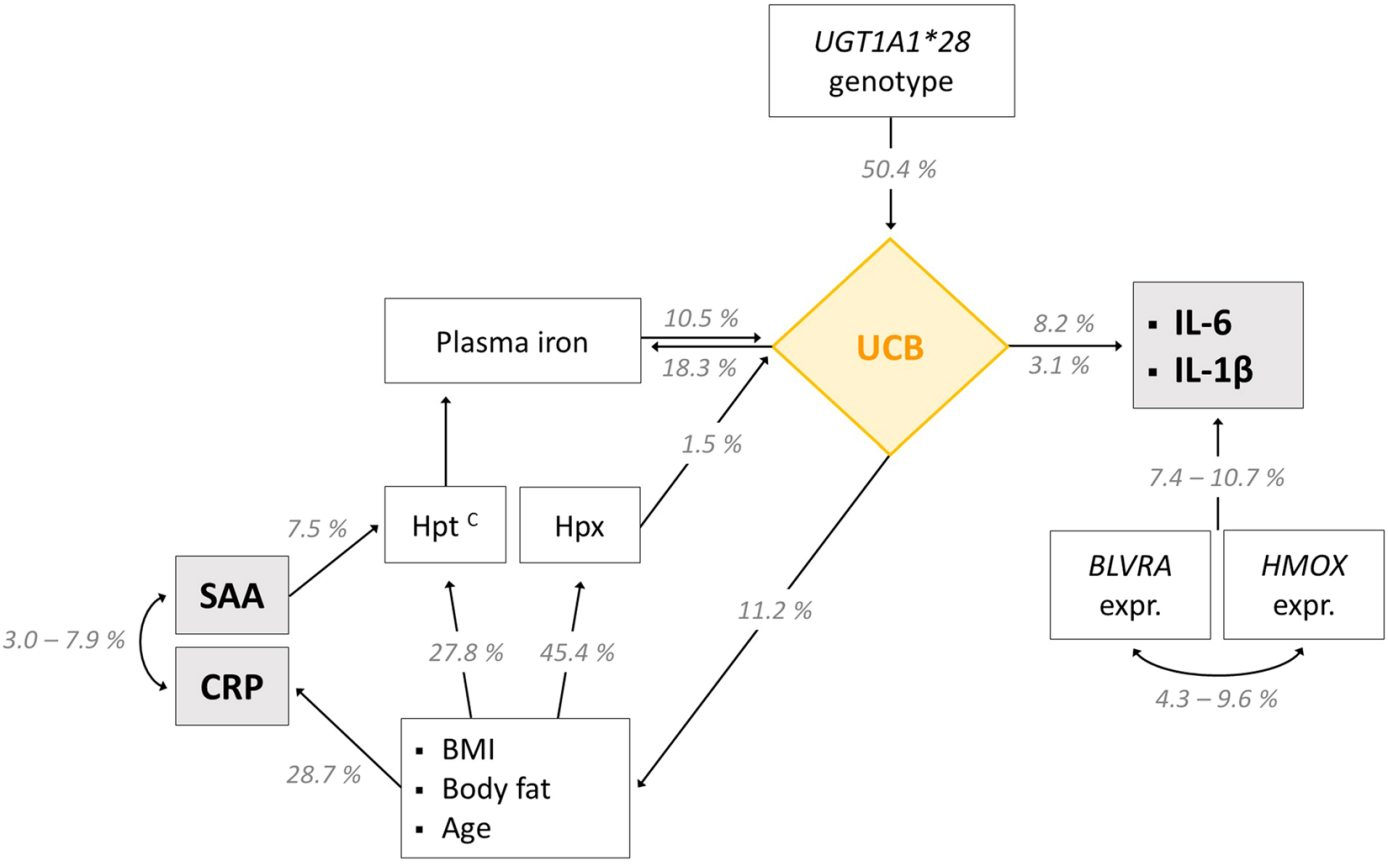
Intracellular levels of interleukins are related to circulating UCB and underlying molecular measures of heme catabolism. Figure 4 shows explanatory powers of parameters that are involved in the heme catabolic pathway for variation in inflammatory markers. Inflammation appears to be directly influenced by UCB on the one hand, and by *HMOX/BLVRA* genetic expressions on the other. As far as UCB was concerned, a further connection with markers of inflammation was found that was mainly established through the variables of body composition (BMI, BF). Variation in the unspecific inflammatory marker of CRP is merely explained by the demographic variable age as well as body composition. The interconnection of iron and UCB emphasizes a potential feedback-loop of UCB on heme catabolism. Stepwise linear regression analysis was performed to assess inter-variable dependence and explanatory power (%), based on corrected R^2^ values and a p-value of ≤0.05 (Supplementary Table S4). ^C^Effect valid for control subjects only. Abbreviations: UCB: unconjugated bilirubin; *UGT1A1*: *UGT1A1* genotype; IL-6/IL-1β: intracellular interleukins 6 and 1β; *BLVRA* expr.: genetic expression of biliverdin reductase; *HMOX* expr.: genetic expression of heme oxygenase; Hpt: haptoglobin; CRP: C-reactive protein; SAA: serum amyloid A.

To further pursue inter-variable connections, and to explore possibilities as to how those entities studied could explain each other on a statistical level, stepwise linear regression models were generated. A graphical abstract of the most relevant findings, can be found in Fig. 4, where percentages (based on corrected R^2^ regression coefficients) specifying significant inter-variable explanatory powers, are presented. Furthermore, a table is provided summarising all relevant correlations that were found (Supplementary Table S4).

Most compelling, and in line with the findings from bivariate correlation analysis, UCB had significant explanatory power for variation in inflammation. Thus, interleukins appeared to be merely dependent on UCB on the one hand and on *BLVRA-/HMOX* genetic expressions on the other, which is also in line with the results obtained from bivariate correlation analysis. Taken together, the explanatory power of these three variables amounted to 18.9%, with UCB explaining 8.2% of variation in IL-6, and 3.1% of variation in IL-1β expression. *BLVRA*- and *HMOX* gene expressions were *vice versa* correlated and explained each other by 9.6 and 4.3%. This outcome was more markedly expressed in C individuals (10.8%).

As expected UCB, the most powerful determinant of interleukin expression not surprisingly was explained by the *UGT1A1* genotype (50.4%). Additional correlated variables included plasma iron (10.5%) and to a smaller extent Hpx (1.5%), readily complementing the findings from bivariate correlation analysis.

Variation in Hpx was furthermore determined by markers of body composition and age (45.4%). The same applied to Hpt (27.8%), and as far as body composition is concerned, also to CRP (28.7%). The latter of which (CRP) was interrelated to SAA with explanatory powers of 3.0 and 7.9%, respectively. The ultimate connector in this interplay of variables once more was UCB, looping back to body composition (6.3 and 4.9%).

## Discussion

The aim of this study was to establish a detailed concept to explain those differences found in GS versus non-GS subjects, concerning improved inflammation, body composition and overall metabolic health. With its numerous intermediate products, the heme catabolic pathway has been extensively studied throughout the past decades. In the present study, a deeper investigation was undertaken, exploring and inter-connecting the molecular pattern that in Caucasians mostly determines the condition of GS (UGT1A1*28) with the genetic background (*HMOX* (GT)_n_) that strongly influences *HMOX* gene and HO-1 enzyme expressions. Specific findings of the study at hand are discussed and summarized in the subsequent paragraphs.

The obtained results provide evidence of an altered heme degradation pathway in the genetic condition of GS, ultimately resulting in a lower inflammatory state. This general finding highlights the comparably lower prevalence and later onset of oxidative stress mediated diseases that is known for individuals with GS^2^. An amplified heme breakdown resulting in UCB-mediated mild hemolysis was indirectly confirmed *1*) by increased UCB levels, the main phenotypic characteristic of GS (rooting in lower *UGT1A1* expression), *2*) by decreased levels of Hpx and Hpt, and *3*) by increased levels of free plasma iron. All of these parameters were found to be relatively elevated in GS versus C individuals. These findings are confirmed by earlier reports^12, 36^. There were no differences in hematocrit and reticulocytes between the groups, confirming the absence of immoderate hemolysis, defects in erythropoiesis and anemia. Also no role for these parameters was found in the correlation models performed. Furthermore, the heme catabolite carbon monoxide (CO, in its measurable form of carbonyl hemoglobin, COHb), had been expected to increase, however, it remained equal between the study groups. Possible reasons and explanations for this surprising result will be discussed further down.

### Heme catabolism and anti-inflammation – implications for improved metabolic health in GS

The phenomenon of a reactively stimulated heme turnover in GS had been reported before^12^, and thus was expected in this study. UCB which is chronically elevated in subjects with GS, is known to loop back to HO-1 activity, as this rate-limiting enzyme of heme breakdown is inducible by heme catabolites on a transcriptional level^12^. The resulting circulating intermediates of heme catalysis – *via* biologically relevant UCB – exert varied effects on inflammation, explaining the observed improved inflammatory state in GS individuals relative to C. On a HO-1 protein level, this inductive property of UCB (and of heme itself) was, however, not confirmed *in vitro* using isolated PBMCs. Here it shall be emphasized that PBMCs were selected as an easily accessible and minimally invasive systemic model to assess the abundance of ubiquitously expressed HO-1^38^ (as opposed to using hepatocytes for instance, which would have been a model of choice). At first glance the above addressed finding of equal inter-group HO-1 levels was surprising, however, can be expected based on the high variation in (GT)_n_-length polymorphisms that was found in this study, differentially encoding for the regulation of the magnitude of HO-1 response to a stress signal^39^ (such as experimental enzyme induction with UCB or heme). A number of recent studies have furthermore shown that specific promoter variations in both the *HMOX-1* and *UGT1A1* gene are responsible for differential *HMOX-1* gene transcription^30, 31^, subsequently influencing HO-1 levels and inducibility.

An alternate explanation that targets HO-1’s nature as a heat-shock protein includes the *in vitro* experimental conditions and their effects on the cells. Following isolation, PBMCs had been freeze-thawed and kept in media overnight. All of which presenting potentially stressful manipulations that likely triggered baseline HO-1 intracellularly, resulting in equal enzyme concentrations between the study groups, and in resistance to further induction by its substrates.

Finally and with reference to the kinetics of transcription (*HMOX-1* gene expression) and translation (HO-1 generation) certainly also the factor of timing should be discussed, since these two steps do not occur simultaneously. Thus it is possible that the exact window of both gene- and protein up-regulation (resulting in a peak in intracellular HO-1 and *HMOX-1* mRNA), was missed.

Upon enzymatic cleavage of heme, equimolar amounts of biliverdin IXα, Fe^2+^ and CO are liberated, eventually resulting in the production of UCB. This molecule has known antioxidant and immune-modulatory properties^16, 19, 40, 41^, whereas Fe^2+^ acts in an anti-microbial fashion^42^ and can be pro-oxidant in the case of Fenton reaction^43^. Further upstream in the pathway, the effects of the heme catabolic enzymes (HO-1, BLVRA) include anti-inflammation as well as they influence NADPH- and oxygen-consuming pathways such as glucose- and lipid metabolism^29^. The observation of an increased *BLVRA* gene expression in males, and the resulting elevated UCB levels might therefore assist in explaining why individuals with GS present with beneficial blood lipid and glucose patterns, relative to non-GS subjects^44, 45^. They are furthermore leaner with lower BMI as has been presented for this and other study collectives^10, 12^. This has ultimate secondary implications for inflammation and associated diseases. In this context, interestingly, *HMOX-1* expression is known to be transcriptionally regulated by PPAR alpha and PPAR gamma^46^, pointing towards a network between heme catabolism and energetic/metabolic regulations with inevitable impact on metabolic health^10^ and secondary inflammation. In the present study, levels of inflammatory cytokines were statistically explained by genetic expressions of the enzymes HO-1 and BLVRA. This finding is readily confirmed by the enzymes’ well-known stress-response properties, exerting cytoprotective, antioxidant, radical-scavenging^17^ and (innate) immune-regulating effects^47^. Why certain phenomena (*e.g*. differential *BLVRA* expression between GS and C) were only found in a gender-specific manner, presently cannot be sufficiently answered. However, sex-specific dimorphic gene expression is a common feature, and has been reported with reference to their implications for the gender-specific prevalence of certain health conditions^48^, likely including GS that is known to more frequently occur in males. The (gender-specific) importance of gene transcriptional activity additionally might have important clinical/therapeutic implications, as for instance the enzyme has been reported to block specific pathways that are involved in the pathogenesis of diabetes^49–52^. The immune-modulating properties of the BLVRA enzyme have furthermore been proposed in innate immunity through exerting an indirect effect on dendritic toll-like-receptor 4^53^ and in allograft acceptance following transplantation^54^.

### UCB and mild hemolysis in heme catabolism

Notably, the heme associates Hpx and Hpt appear to be significantly involved in the process of heme degradation with a confirmed increase in UCB, decrease in inflammation and improved body composition. The association with UCB is specifically meaningful as a decrease in Hpt is sensitively connected to hemolysis. This pursues the suggested theory of mild hemolysis in GS^12, 36, 37^, resulting from a mildly increased UCB-driven breakdown of erythrocytes^12^. Reversely, this mild form hemolysis, however, is said to be insufficient to produce hyperbilirubinemia *per se* ^37, 55, 56^. In connection to this, the feature of additional diserythropoiesis has been suggested for the condition of GS^36^, which was not confirmed in the present study, based on equal reticulocyte counts between the groups.

In addition to hemolysis other Hpx and Hpt effects include tissue protection through high-affinity binding of free heme, as unbound heme when present in excess is known to exert its pro-oxidant effects on tissues^25^. Thus this feature subsequently serves anti-inflammation, as was observed in GS individuals.

### The “CO-dilemma”

The physiological function of CO remains unclear. It is generally accepted that CO at high concentrations is toxic to cells, but less is known about pico-levels in physiological function^20^. There is evidence that CO plays a vital role in modulating cellular bioenergetics^29^ and its generation within heme catabolism has been linked to antioxidant, immune-modulating^47^ as well as anti-atherosclerotic activities^11^. In this respect, a physiological increase of CO had been found previously by our group in the condition of GS^12^, and thus represents a phenomenon that is known to occur when heme catabolism is reactively boosted. It was therefore unexpected and somewhat surprising that in the present study COHb levels remained equal between the study groups.

Based on its antioxidant nature, CO is a sensitive marker readily reflecting changes in the cellular redox equilibrium. Thus, it is known to rise upon (active) exposure to tobacco smoke, and other environmental noxae including combustion of fossil fuels (*e.g*. while home gas cooking or during the heating period)^57^. Therefore, marked inter-individual and seasonal fluctuations in COHb are possible and in fact to be expected. As the study was performed over the duration of eight months, those potential differences in COHb present could have been disguised by natural fluctuations. Despite the fact that all subjects were self-reported non-smokers, certainly, also the main CO-influencing factor of (secondary) cigarette smoking cannot be reliably excluded.

Though CO is known to rise when heme catabolism is boosted, care should be taken, when inversely assessing the catalytic activity of HO-1 solely based on concentration of CO, as increased CO formation was previously described to occur in an HO-independent fashion as a result of photo-oxidation^58^. Therefore, the crucial factor of fast sample processing and accurate handling needs to be taken into account, as this can substantially influence results.

If or to what extent those potential influencers of CO formation have been relevant in the present study remains unclear and can only be speculated about. Especially when taking into account that in both groups COHb levels were generally low.

After all, while it is generally accepted that CO may collaborate with UCB in achieving physiological effects, in numerous studies the latter has been shown to be the leading mediator of HO-1’s protective actions^15^.

### The *HMOX* (GT)_n_ genotype in regulating HO-1 and its possible implications for health

At this particular time, a clear role for the *HMOX* microsatellite genotype in the heme degradation process cannot be derived from this study’s findings. Based on the various allelic combinations this genotype can adopt, likely a larger study collective would be needed to obtain significant results. Therefore, (GT)_n_-repeats were statistically not able to definitely explain certain regulatory aspects of the heme catabolic pathway that are inherent to the condition of GS. However, as a correlated variable the GT genotype was negatively connected to BMI, body fat, *HMOX-*1 and *BLVRA* gene expressions, and most importantly to the inflammatory markers of IL-6, IL-1β, CRP and SAA. These results are clearly in line with the literature concerning the negative associations of shorter GT-repeats with anti-inflammation and UCB^34, 59–62^, and emphasize a potential role for heme catabolism and its players in disease prevention, as a “therapeutic funnel”^39^. Once the multifaceted molecular influence of underlying genotypes (*HMOX-1* (GT)_n_, *UGT1A1*) on HO-1 function has been clarified, prior enzymatic induction or administration of heme intermediates in pathologic conditions of overwhelming stress circumstances could be potentially favourable^63, 64^.

## Conclusion

The study at hand confirms those phenotypic particularities that are known from the literature for GS individuals, concerning inflammation and overall metabolic health. As a causative factor, an altered heme catabolism was indirectly determined in the present study, reflecting in significantly elevated UCB (the main phenotypic characteristic of GS rooting in lower *UGT1A1* transcription) and iron levels, as well as in decreased concentrations of Hpx and Hpt. The latter of which strongly supporting the theory of mild UCB-mediated hemolysis, that has been suggested for the condition of GS^12^. Surprisingly, other heme related parameters such as COHb, Hb and reticulocyte counts remained unchanged between the groups.

A clear role for the underlying *HMOX* (GT)_n_ genotype in fine-tuning the heme catabolic pathway to date cannot be reliably derived from the present findings. However, a specific inter-group pattern in allelic distribution of GT fragment lengths was observed, although this result did not reach statistical significance, likely based on group size. Nevertheless, this result is readily supported by previous findings from the literature concerning the association of shorter GT microsatellites with increased UCB levels^34, 59–61^ as well as with decreased inflammation^34, 62^, possibly in ultimate response to an increase in UCB.

The significantly lower degree of inflammation confirmed for the GS group is particularly remarkable, since all individuals (GS and C) were healthy and free of any underlying (chronic) diseases. The decreased inflammatory status might be key towards explaining the lower prevalence and later onset of severe oxidative-stress mediated diseases in GS.

## Materials and Methods

### Subjects and study design

This study (abbreviated “BiliHealth”) was designed as an observational case-control study, at a single centre in Vienna, Austria. The study was performed at the Department of Clinical Pharmacology at the Vienna General Hospital. Subjects with and without the condition of GS were recruited between June 2014 and January 2015, by direct advertising (bulletin boards, posters and flyers) and from the department’s subject database.

One hundred twenty-eight (128) healthy subjects of Caucasian ancestry between 20 and 80 years of age were initially recruited from the general Austrian population. Eight thereof had to be excluded for medical or lifestyle reasons which only became apparent upon their initial screening examination (supplement intake, medication, elevated liver enzymes, smoking). Exclusion criteria included excess drinking, smoking, excess physical activity, routine intake of medications and nutritional supplements, pregnancy, acute and chronic (inflammatory/metabolic) diseases, liver diseases/elevated liver enzymes, present or past neoplasia and organ transplants. After providing their signed informed written consent form, each subject completed an initial health check-up (fasting blood biochemistry including levels of unconjugated bilirubin (UCB) and liver enzymes, blood pressure, body weight/-height, lifestyle questionnaires).

A total of 80 males and 40 females completed the study. This gender distribution is representative of the occurrence of GS in the general population^65^. All subjects were age- and gender-matched, and study group allocation (GS, C) was based on the subjects’ respective fasting serum UCB concentrations (</≥ 17.1 µM)^65^, that had been analysed using HPLC. For the most part, subjects with GS (in contrast to C) showed visible signs of mild jaundice, reflecting in a yellowish pigmentation of the skin and the conjunctival membranes over the sclerae. Liver parameters and parameters of severe hemolysis were within the normal ranges. Participants were furthermore allocated to age groups (</≥ 35 years of age). A graphical summary of the study design was published previously^10^, and can be found in the online supplement of that report.

For the purpose of diagnosing GS, all subjects of both study groups were required to fast on the day before participating in the study, and therefore had to follow a 400 kcal fasting protocol^1, 12^. Furthermore, a complete overnight fast of 16 (±1) hours was required, before the day of blood sampling (study day).

Characteristics of the study population, including age distribution, UCB levels and aspects of lifestyle, are summarized in Supplementary Table S1.

### Ethics

This study was approved by the Ethics Commission of the Medical University of Vienna (No. 1164/2014), and was conducted in accordance with the approved guidelines by the Declaration of Helsinki.

### Blood biochemistry (whole blood, plasma, serum)

For each subject, fasting blood samples were collected on a single occasion (baseline), no longer than two weeks from the entry health check-up. Samples were drawn by venepuncture into EDTA, Li-Heparin and serum tubes (K_2_EDTA, Li-Heparin and Z Serum Sep, respectively). Samples were cooled and protected from light until being analysed or aliquoted. Aliquots were stored at −80 °C until further analysis.

Besides UCB, liver enzymes (aspartate aminotransferase, AST; alanine transaminase, ALT; gamma-glutamyl trans peptidase, γ-GT; lactate dehydrogenase, LDH), ferritin, transferrin, hormones (thyroid stimulating hormone, TSH; triiodothyronine, T3; thyroxin, T4), a range of lipid parameters (total cholesterol, TChol; high density lipoprotein, HDL; low density lipoprotein, LDL; triglycerides, TG; ApoA1, apolipoprotein A1; ApoB, apolipoprotein B; lipoprotein A2, LPA2) and differential blood counts were automatically analysed in the routine central laboratories of the Vienna General Hospital (Olympus 5400 clinical chemistry analysers, Beckman Coulter). Hemoglobin and reticulocyte counts were analysed in EDTA whole blood using an automated hematology system (Sysmex Hematology Systems, Inc., Canada). Carbonyl hemoglobin (COHb) was measured directly from heparinized syringes, using a blood gas analyser ABL 700 (Radiometer, Brønshøj, Denmark). C-reactive protein (CRP) was analysed using the high-sensitive CRP Latex immune-turbidimetric assay (Olympus 5400 clinical chemistry analysers, Beckman Coulter).

All parameters were measured on the day of blood sampling. Not all of the parameters were selected for presentation in this publication.

### UCB measurement (HPLC) in serum

For a detailed analysis of UCB (isomers), HPLC was conducted (after Brower *et al*.^66^), as had been used and published by our group^9, 10, 12^ and others^67^ previously. Samples were protected from light exposure and kept at 4 °C throughout all processing steps. Briefly, fasting serum samples (stored in amber vials) were diluted in isocratic mobile phase (methanol, water, *n-*dioctylamine and acetic acid) and centrifuged. Supernatants were run on a chromatograph (Merck, Hitachi, LaChrom), equipped with a photodiode array detector (PDA, Shimadzu) and a Fortis C18 HPLC column (4.6 × 150 mm, 3 µm), with a Phenomenex C18 HPLC guard column (4 × 3 mm). Sample preparation and analysis followed the previously published protocol^12^. For the purpose of quality control unconjugated bilirubin (Frontier Scientific Europe, Carnforth, Lancashire, UK) with an isomeric purity of >99% served as an external standard. Additionally, to assure consistency in analyte recovery a reference serum sample was run in each analysis.

### Anthropometric measurements

Standing height (subjects without shoes and in relaxed upright position) was measured with a commercial stadiometer, to the nearest 0.5 cm. Body mass (subjects barefooted and lightly dressed) was assessed to the nearest 0.1 kg, using digital scales. The body mass index (BMI) was calculated following the equation BMI = body mass [kg]/(body height^2^ [m^2^]). To determine body composition, Bioelectric Impedance Analysis (BIA) was used, providing reliable data of body composition^68^, and was performed in the mornings of the study days, using a BIA Analyser 2000-S (Data-Input GmbH, Darmstadt, Germany).

### Flow cytometric measurement of interleukins in whole blood (IL-6, IL-1β, TNFα)

IL-6, IL-1b and TNFα were measured in monocytes (CD14^+^) following the standard intracellular protein staining protocol (BD) using a FACS Calibur (BD) flow cytometer. Briefly, heparinized whole blood was treated with brefeldin A solution (BD) for 4 h, to block cytokine excretion from cells. Erythrocytes were lysed (Cell Lysis Solution 1:10, BD), removed, and white cells were stained with monocyte-specific anti-CD14-APC surface antibody (Biolegend). Where applicable (positive controls), LPS stimulation was performed over 4 h, using bacterial LPS from E. coli (Sigma) at a final concentration of 1 µg/mL. In the next step, cells were permeabilised (Permeabilising Solution 2, 1:10, BD), and treated with intracellular anti-IL-6-PE, anti-IL-1b-FITC and anti-TNFα-PerCP-Cy5.5 antibodies (BD). Antibodies had been titrated before use. Cells were measured immediately following staining and relative fluorescence intensity was quantified against isotype control. For quality control, Hick-3 cytokine positive control cells (BD) were used in every run, as well as signal compensation had been successfully performed prior to each analysis.

### Flow cytometric (FACS) analyses of HO-1 in PBMCs (baseline)

Intracellular protein concentrations were measured in peripheral blood mononucleated cells (PBMCs). Cells were extracted from EDTA whole blood immediately after blood samplings. Density gradient centrifugation using separation tubes (Leucosep^TM^, Greiner bio one GmbH, Austria) was applied as instructed. Following isolation, cells were washed twice with ice-cold PBS. Cell count and viability were assessed using the trypan blue exclusion assay on an automated cell counter (Countess^TM^, Life Technologies). For short-term storage, cells were aliquoted in freezing medium (FBS + 10% DMSO) and gradually cooled (1 °C/min) to −80 °C, using the CoolCell^TM^ system (Biozym).

All FACS analyses were completed on a four-channel FACS Calibur^TM^ flow cytometer (BD, Europe). Signal compensation (using Calibrite^TM^ beads and FACS Comp software, BD) was successfully completed prior to each experimental run.

PBMCs were thawed at 37 °C and washed twice with cold PBS (3000 g, 5 min). Cell count per test tube was adjusted to 250.000. Cells were kept overnight in supplemented RPMI medium^69^. After washing, cells were fixed (1% formalin, 10 min, RT), washed again, and permeabilised (70% ice-cold ethanol, 10 min, on ice). Following another washing step, cell pellets were suspended in staining-buffer (1% BSA, 0.02% Na-azide in PBS), and stained with PE-anti-HO1/2 antibody (30 min, on ice; ab83214 Abcam; where applicable). The antibody had been titrated before use. Samples were run twice as independent duplicates, relative to respective negative/isotype controls. Fluorescence signals (relative fluorescence units, rfU) were detected and recorded in the respective channels, and compared between the study groups.

### Flow cytometric (FACS) analyses of HO-1 in PBMCs (following bilirubin or heme incubation)

PBMCs were thawed at 37 °C and washed twice with cold PBS (3000 g, 5 min). Cell count per test tube was adjusted to 250.000. Cells were incubated with 70 µM unconjugated bilirubin alpha or 150 µM heme (both Sigma) overnight. Potential cytotoxic UCB and heme effects had been excluded through a viability check (trypan blue assay). Bile pigments were delivered to cells in DMSO into supplemented RPMI medium (according to Jeon *et al*.^69^). Final DMSO concentrations did not exceed 0.1%. After washing twice, cells were fixed (1% formalin, 10 min, RT), washed again, and permeabilised (70% ice-cold ethanol, 10 min, on ice). Following another washing step, cell pellets were suspended in staining-buffer (1% BSA, 0.02% Na-azide in PBS), and stained with PE-anti-HO1/2 antibody (30 min, on ice; ab83214 Abcam; where applicable). The antibody had been titrated before use. Samples were run twice as independent duplicates, relative to respective negative/isotype controls. Fluorescence signals (relative fluorescence units, rfU) were detected and recorded in the respective channels, and compared between the study groups.

### *UGT1A1* Genotyping (-TA repeats in UGT1A1*28 promoter region)

For *UGT1A1* genotyping purposes, DNA was extracted from whole blood, using a QIAsymphony SP automated system with QIAsymphony DSP DNA Midi Kit (QIAGEN), as instructed.

Analyses were performed as described elsewhere^70^. Primers and probes were used as 10 µM working solutions. LightCyclerFastStart DNA Master HybProbe Mix (Roche) was used on a LightCycler 480 Instrument II (Roche). Alleles were determined according to the melting curves obtained.

### *HMOX-1* Genotyping (-(GT)n repeats/fragment length)

DNA for *HMOX-1* genotyping was extracted from whole blood, using the QIAsymphony SP automated system with QIAsymphony DSP DNA Midi Kit (QIAGEN), according to the manufacturer’s instructions.

The method was carried out according to Mustafa *et al*.^71^ (with modifications). Briefly, determination of the GT-repeat located in the 5′-flanking region of the *HMOX* gene (g.15167397GT (23_37); dbSNP rs3074372) was done by PCR, using a fluorescently labeled forward primer: 5′-FAM-AGAGCCTGCAGCTTCTCAGA-3′ and non-labelled reverse primer: 5′-ACAAAGTCTGGCCATAGGAC-3′. PCR was conducted using Platinum Taq DNA polymerase (Life Technologies), 0.863 mmol/L MgCl2, 200 µmol/L of each dNTP (Sigma-Aldrich), 4 pmol forward primer, 4 pmol reverse primer, and approximately 4 ng DNA. Amplifications were performed in 96-well plates on an ABI 7300 cycler (Applied Biosystems). The cycling program was as follows: 5 minute polymerase activation at 94 °C; 35 cycles of 94 °C for 30 s denaturation, 58 °C for 15 s annealing, 72 °C for 30 s extension. A final extension step of 60 minutes at 72 °C was performed at the end. The length of the PCR products was determined relative to an internal size-standard (GeneScan ROX 350 size standard, Applied Biosystems), after 5 minutes denaturation at 95 °C in the presence of 10 µL formamide (Sigma-Aldrich) per 1.5 µL PCR reaction, on an automated DNA capillary sequencer (ABI Prism 3100 Genetic Analyser; Applied Bio-systems). Fragment length determination and GT-repeat length attribution was done semi-automatically using ABI Prism Software (Gene Scan Analysis Version 3.7 and Genotyper Software Version 3.7, Applied Biosystems). Classification of GT-fragment lengths (S < 26, L > 30) was performed following Chang *et al*.^72^.

### RNA extraction, cDNA synthesis and qPCR of *HMOX-1 and BLVRA* gene expression

The starting material for RNA extraction were PBMCs, isolated from whole blood. RNA extraction was performed with the Qiagen RNeasy® Mini Kit, as instructed by the manufacturer, and using the QIAcube automated system. Total RNA concentration and quality were estimated using NanoDrop® ND-1000 (Thermo Scientific). Reverse transcription RNA to cDNA was performed using the High-Capacity cDNA Reverse Transcription Kit with RNase Inhibitor 1000 Reactions (Applied Biosystems) on a Biometra thermocycler. The quality and quantity of cDNA first strand were estimated by NanoDrop 2000c spectrophotometer. Single-plex commercially available TaqMan assays (Life Technologies) were performed with 10 ng cDNA as a template. The TaqMan assay for *BLVRA* (assay Hs00167599_m1, FAM-MGB-labelled) and *HMOX-1* (Hs01110250_m1, FAM-MGB-labelled) genes was used according to manufacturer instructions. *ACTB* (assay Hs99999903_m1) and *GAPDH* (Hs99999905_m1) were used as endogenous controls. The PCR amplification was completed using TaqMan Universal PCR master mix on a QuantStudio™ 6 Flex Real-Time PCR System (Thermo Fisher), using a 384-well block. All samples were run on the same 384-well plate in a single run, to avoid inter-plate variations. All samples were tested in triplicate, and samples with a standard deviation higher than 0.5 Ct-units were excluded from further analyses. Relative quantification was done using the RQ (Relative Quantification) feature on the Thermo Fisher Cloud qPCR analysis software with *ACTB* and *GAPDH* as endogenous controls and a pooled cDNA sample as a reference.

### Statistical analyses

Statistics were completed using IBM SPSS 24 [IBM Corp. Released 2016. IBM SPSS Statistics for Windows, version 24.0. Armonk, NY: IBM Corp.]. Data distribution was checked using Kolmogorov-Smirnov (K-S test) and histograms. For comparison of means (for parametric data), ANOVA was used, for comparing medians or ranks (for non-parametric data), Mann-Whitney-U-test was selected. Data were summarized as was appropriate according to their respective distribution. For parametric data, means ± sd (standard deviation), for non-parametric variables, medians ± IQR (inter-quartile range) are presented. To display frequencies, crosstabs and histograms were selected. Bivariate correlations were modelled using Spearman correlation analysis (Spearman’s *rho*). Correlation coefficients (R) and p-values are presented. Regressions were calculated by applying the model of stepwise linear regression. Corrected R^2^ coefficients and p-values are presented. For all statistical measures, the level of significance was set to be 5% (p ≤ 0.05). Trends were defined on a 10% level of error (p ≤ 0.1).

## Electronic supplementary material

Supplementary Information
