# Supplementary material for: Characteristics of the heme catabolic pathway in mild unconjugated hyperbilirubinemia and their associations with inflammation and disease prevention
Source: Sci Rep. 2017 Apr 7;7:755. doi: 10.1038/s41598-017-00933-y (PMC5429724; doi:10.1038/s41598-017-00933-y)
Supplement: Supplementary file 1 — Supplementary Information [file 41598_2017_933_MOESM1_ESM.pdf]

Characteristics of the heme catabolic pathway in mild unconjugated hyperbilirubinemia and their associations with inflammation and disease prevention

Christine Mölzer, Marlies Wallner, Carina Kern, Anela Tosevska, René Zadnikar, Daniel Doberer, Rodrig Marculescu, Karl-Heinz Wagner

SUPPLEMENTARY FIGURES AND TABLES:

<u>Supplementary table S1.</u> Demographical description of the BiliHealth study population (males and females), including the key parameters UCB and heme.

Variables	Males		*p-value*	Females		*p-value*
	GS	***C***		***GS***	***C***	
Subjects [n]	40	40	*1.000*	20	20	*1.000*
Median age [yrs]^	30 (19)	31 (20)	*0.965*	40 (20)	40 (18)	*0.766*
Subjects aged </> 35 yrs [n/n]	24/16	24/16	*1.000*	9/11	9/11	*1.000*
Age of subjects </> 35 yrs^	26.5 (6.0)/45.5 (15.0)	27.0 (6.0)/45.5 (14.0)	*0.000*	29 (5)/48 (19)	29 (5)/46 (14)	*0.000*
BMI [kg/m^2]^/°	22.5 (3.6)	24.4 (5.9)	*0.023**	21.8 (± 2.7)	24.7 (± 4.4)	*0.017**
Body fat [%]^/°	20.7 (7)	19.2 (9)	*0.838*	24.4 (± 6.9)	30.8 (± 7.8)	*0.011**
UCB concentration [µM]°	35.3 (± 10.4)	9.7 (± 3.5)	*0.000**	28.9 (± 6.8)	8.4 (± 3.1)	*0.000**
*UGT1A1*28* genotype/-TA repeats [% 7_7/6_7/6_6]□	86.8/10.5/2.5	5.3/50/44.7	*0.000**	94.7/5.3/0	5.3/45/45	*0.000**
Heme [µM]^	0.70 (0.10)	0.75 (0.10)	*0.088*^{T}	0.71 (0.20)	0.72 (0.20)	*0.826*
Intermediate blood pressure [RR, mm Hg]°	102 (± 10)	104 (± 11)	*0.425*	92 (± 10)	96 (± 12)	*0.354*
AST [U/L]^	26 (7)	25 (11)	*0.932*	22 (9)	21 (3)	*0.397*
ALT [U/L]°/^	24 (± 7)	26 (± 9)	*0.328*	20 (14)	19 (8)	*0.944*
GGT [U/L]^	19 (17)	21 (16)	*0.838*	11 (4)	14 (5)	*0.082*^{T}
LDH [U/L]^/°	158 (30)	156 (42)	*0.561*	164 (± 29)	159 (± 35)	*0.676*
Albumin [mg/mL]°	48.1 (± 3.4)	47.0 (± 3.2)	*0.154*	45.2 (± 2.2)	45.8 (± 1.8)	*0.362*

Based on data distribution, means° or medians^ are presented. For parametric data mean ± sd, for non-parametric distribution, median and IQR (inter-quartile range) are given. P-values of ≤ 0.05* indicate significant differences; trends are reflected by p ≤ 0.1^{T}.

□Insertion of additional –TA repeats in the *UGT1A1*28* promoter region; 7_7: Gilbert's syndrome, 6_7: heterozygous individuals, 6_6: wild type.

<u>Abbreviations</u>: BMI: Body mass index; UCB: unconjugated bilirubin; *UGT1A1*-genotype: UDP glucuronosyltransferase 1A1 genotype; AST: Aspartate aminotransferase; ALT: Alanine aminotransferase; GGT: Gamma-glutamyl transferase; LDH: Lactate dehydrogenase

Supplementary table S2. **Parameters of the heme catabolic pathway, immunology and hematology, including male subjects from the BiliHealth study.**

MALE	Variable	Mean° (± sd)/median^ (IQR) GS	C	*p-value*
Heme catabolic parameters	*HMOX* expr. [RQ]^	0.96 (0.32)<br>n = 37	0.92 (0.32)<br>n = 36	*0.242*
	HMOX genotype/GT-repeats [bp]^	27.5 (3.5)<br>n = 37	28.8 (3.5)<br>n = 36	*0.955*
	HO-1/2 BL [rfU]°	195 (± 78)<br>n = 40	206 (± 82)<br>n = 40	*0.570*
	HO-1/2 induced (BR) [x-fold]^	0.35 (0.4)<br>n = 40	0.34 (0.5)<br>n = 39	*0.576*
	HO-1/2 induced (H) [x-fold]^	0.12 (0.3)<br>n = 40	0.06 (0.2)<br>n = 39	*0.439*
	BLVRA expr. [RQ]^	0.83 (0.22)<br>n = 38	0.67 (0.25)<br>n = 37	*0.038**
Immunological and hematological parameters	IL-6 BL [rfU]°	2.3 (± 0.2)<br>n = 37	2.6 (± 0.3)<br>n = 39	*0.000**
	IL-1β BL [rfU]°	1.4 (± 0.2)<br>n = 37	1.6 (± 0.3)<br>n = 39	*0.002**
	TNFα BL [rfU]°	25.4 (± 5.9)<br>n = 38	26.2 (± 5.2)<br>n = 39	*0.536*
	IL-6 induced [x-fold]°	8 (± 4)<br>n = 37	7 (± 4)<br>n = 39	*0.392*
	IL-1β induced [x-fold]^	21 (13)<br>n = 36	18 (8)<br>n = 39	*0.142*
	TNFα induced [x-fold]^	9 (11)<br>n = 38	8 (8)<br>n = 38	*0.720*
	CRP [mg/dL]^	0.05 (0.05)<br>n = 37	0.07 (0.09)<br>n = 36	*0.405*
	Hemopexin [mg/dL]°	81 (± 10)<br>n = 32	85 (± 15)<br>n = 37	*0.243*
	Haptoglobin [mg/dL]^	89 (49)<br>n = 32	90 (59)<br>n = 37	*0.485*
	Plasma iron [µg/dL]°	180 (± 60)<br>n = 39	130 (± 50)<br>n = 39	*0.000**
	Ferritin [µg/L]^	126 (120)<br>n = 39	129 (90)<br>n = 40	*0.855*
	Hematocrit [%]^	45 (4)<br>n = 39	43 (4)<br>n = 40	*0.070^T*
	COHb [%]^	1.20 (0.20)<br>n = 40	1.20 (0.50)<br>n = 40	*0.911*
	SAA [mg/L]^	3.9 (0.0)<br>n = 39	3.9 (0.2)<br>n = 39	*0.537*
	Uric acid [mg/dL]°	5.9 (± 0.9)<br>n = 40	5.7 (± 1.0)<br>n = 40	*0.460*

Values are specified as applies according to distribution of data. For parametric variables, means° ± sd are shown, for non-parametric data, medians^ (50th percentiles) and inter-quartile range (IQR) are displayed. Comparison of means for parametric data or of ranks (for non-parametric data) was completed using independent samples t-test or Mann-Whitney-U-test. *p-value: significant on a 5 % level of significance; ^{T}p-value: trend on a 10 % level of trend.

Abbreviations: GS: Gilbert's syndrome; C: Controls; *HMOX* expr. [RQ]: Heme oxygenase gene expression as relative quantification [RQ to cDNA pool]; [bp] base pairs; HO-1/2 [rfU]: intracellular heme oxygenase in PBMCs [relative fluorescence]; HO-1/2 induced (BR)/(H): fold-increase in intracellular heme oxygenase, induced by incubation with unconjugated bilirubin (BR) or heme (H) overnight, relative to baseline; *BLVRA* expr.: Biliverdin reductase gene expression as relative quantification [RQ to cDNA pool]; IL-6 BL/IL-1β BL/TNFα BL [rfU]: baseline (BL) intracellular (PBMCs) levels of interleukins; IL-6 induced/IL-1β induced/TNFα induced [x-fold]: fold-increase in intracellular (PBMCs) levels of interleukins upon LPS-stimulation, relative to baseline; CRP: C-reactive protein; COHb: Carbonyl hemoglobin; SAA: Serum amyloid A.

Supplementary table S3. Parameters of the heme catabolic pathway, immunology and hematology, including female subjects from the BiliHealth study.

FEMALE	Variable	Mean° (± sd)/median^ (IQR) GS	C	*p-value*
Heme catabolic parameters	*HMOX* expr. [RQ]°	0.99 (± 0.24) n = 16	1.02 (± 0.34) n = 15	*0.777*
	HMOX genotype/GT-repeats [bp]^	27.0 (3.5) n = 19	30.0 (3.0) n = 19	*0.276*
	HO-1/2 BL [rfU]°	197 (± 61) n = 20	210 (± 51) n = 20	*0.462*
	HO-1/2 induced (BR) [x-fold]°	0.4 (± 0.4) n = 19	0.5 (± 0.3) n = 20	*0.628*
	HO-1/2 induced (H) [x-fold]^	0.06 (0.2) n = 19	0.12 (0.2) n = 20	*0.433*
	BLVRA expr. [RQ]°	0.83 (± 0.2) n = 16	0.86 (± 0.19) n = 17	*0.646*
Immunological and hematological parameters	IL-6 BL [rfU]°	2.4 (± 0.3) n = 20	2.5 (± 0.3) n = 20	*0.410*
	IL-1β BL [rfU]^	1.3 (0.5) n = 20	1.4 (0.3) n = 20	*0.766*
	TNFα BL [rfU]°	26.4 (± 4.7) n = 20	25.1 (± 4.8) n = 20	*0.421*
	IL-6 induced [x-fold]°	7 (± 4) n = 20	8 (± 5) n = 20	*0.205*
	IL-1β induced [x-fold]°	22 (± 11) n = 20	25 (± 11) n = 20	*0.363*
	TNFα induced [x-fold]^	3 (7) n = 19	10 (16) n = 20	*0.044**
	CRP [mg/dL]^	0.03 (0.06) n = 20	0.07 (0.07) n = 18	*0.019**
	Hemopexin [mg/dL]°	88 (± 7) n = 19	92 (± 12) n = 20	*0.196*
	Haptoglobin [mg/dL]°	62 (± 25) n = 19	109 (± 51) n = 20	*0.001**
	Plasma iron [µg/dL]^	149 (56) n = 19	113 (36) n = 18	*0.025**
	Ferritin [µg/L]^	35 (35) n = 19	48 (78) n = 20	*0.286*
	Hematocrit [%]°	38 (± 2) n = 20	38 (± 3) n = 20	*0.890*
	COHb [%]^	1.10 (0.30) n = 20	1.10 (0.30) n = 20	*0.869*
	SAA [mg/L]^	3.9 (0.0) n = 19	4.3 (1.4) n = 18	*0.009**
	Uric acid [mg/dL]°	4.4 (± 0.9) n = 20	4.2 (± 0.9) n = 20	*0.546*

Values are specified as applies according to distribution of data. For parametric variables, means° ± sd are shown, for non-parametric data, medians^ (50th percentiles) and inter-quartile range (IQR) are displayed. Comparison of means for parametric data or of ranks (for non-parametric data) was completed using independent samples t-test or Mann-Whitney-U-test. *p-value: significant on a 5 % level of significance; ^{T}p-value: trend on a 10 % level of trend.

Abbreviations: GS: Gilbert's syndrome; C: Controls; *HMOX* expr. [RQ]: Heme oxygenase gene expression as relative quantification [RQ to cDNA pool]; [bp] base pairs; HO-1/2 [rfU]: intracellular heme oxygenase in PBMCs [relative fluorescence]; HO-1/2 induced (BR)/(H): fold-increase in intracellular heme oxygenase, induced by incubation with unconjugated bilirubin (BR) or heme (H) overnight, relative to baseline; *BLVRA* expr.: Biliverdin reductase gene expression as relative quantification [RQ to cDNA pool]; IL-6 BL/IL-1β BL/TNFα BL [rfU]: baseline (BL) intracellular (PBMCs) levels of interleukins; IL-6 induced/IL-1β induced/TNFα induced [x-fold]: fold-increase in intracellular (PBMCs) levels of interleukins upon LPS-stimulation, relative to baseline; CRP: C-reactive protein; COHb: Carbonyl hemoglobin; SAA: Serum amyloid A.

<u>Supplementary table S4.</u> Stepwise linear regression analysis for key variables included in figure 4.

**DEPENDENT VARIABLES:**	*UCB*	*UGT1A1*	*BMI*	*BF*	*CRP*	*HMOX gene expr.*	*BLVRA gene expr.*	*SAA*	*Age*	*Hpx*	*Plasma iron*	***Model summary***
IL-6	0.082 *(0.003)*					0.030 *(0.000)*	0.077 *(0.000)*					0.189 *(0.000)*
IL-1β	0.031 *(0.002)*						0.074 *(0.004)*					0.105 *(0.002)*
CRP			0.287 *(0.000)*					0.030 *(0.000)*				0.317 *(0.000)*
SAA					0.079 *(0.003)*							0.079 (0.003)
Hpx				0.343 *(0.000)*					0.111 *(0.000)*			0.454 *(0.000)*
Hpt			0.253 *(0.000)*					0.075 *(0.000)*	0.025 *(0.000)*			0.353 *(0.000)*
UCB		0.504 *(0.000)*								0.015 *(0.000)*	0.105 *(0.000)*	0.624 *(0.000)*
Plasma iron	0.183 *(0.000)*											0.183 *(0.000)*
HMOX gene expr.							0.043 *(0.027)* 0.108 *(0.013)*^{C}					0.043 *(0.027)* 0.108 *(0.013)*^{C}
BLVRA gene expr.						0.096 *(0.003)* 0.108 *(0.013)*^{C}						0.096 *(0.003)* 0.108 *(0.013)*^{C}
BMI	0.063 *(0.000)*								0.104 *(0.001)*			0.167 *(0.000)*
BF	0.049 *(0.000)*								0.101 *(0.001)*			0.150 *(0.000)*

Corrected R^2 coefficients and corresponding p-values *(in brackets)* from stepwise linear regression analysis are provided.
Unspecified regressions are valid for the entire study population. ^{C} Effect valid for control subjects only.

<u>Abbreviations:</u> IL-6: intracellular interleukin 6; IL-1β : intracellular interleukin 1β; CRP: C-reactive protein; SAA: serum amyloid A; Hpx: hemopexin; Hpt: haptoglobin; UCB: unconjugated bilirubin; *HMOX* gene expression: gene expression of heme oxygenase 1; *BLVRA* gene expression: gene expression of biliverdin reductase; BMI: body mass index; BF: body fat.

<u>Included variables (dependent variables in bold):</u>
IL-6: age, BMI, UCB, *HMOX*-genotype, *HMOX* GT-repeats, *HMOX* gene expression, *BLVRA*, gender, BF, CRP, SAA, Iron.
IL-1β: age, BMI, UCB, *HMOX*-genotype, *HMOX* GT-repeats, *HMOX* gene expression, *BLVRA*, gender, BF, CRP, SAA, Iron.
CRP: age, BMI, UCB, *HMOX*-genotype, *HMOX* GT-repeats, *HMOX* gene expression, *BLVRA*, gender, BF, SAA, Iron, IL-6, IL-1β, TNF.
SAA: age, BMI, UCB, *HMOX*-genotype, *HMOX* GT-repeats, *HMOX* gene expression, *BLVRA*, gender, BF, CRP, Iron, IL-6, IL-1β, TNF.
Hpx: age, BMI, UCB, *HMOX*-genotype, *HMOX* GT-repeats, *HMOX* gene expression, *BLVRA*, gender, BF, SAA, Iron, IL-6, IL-1β, TNF.
Hpt: age, BMI, UCB, *HMOX*-genotype, *HMOX* GT-repeats, *HMOX* gene expression, *BLVRA*, gender, BF, SAA, Iron, IL-6, IL-1β, TNF.
UCB: age, BMI, *HMOX*-genotype, *HMOX* GT-repeats, *HMOX* gene expression, *BLVRA*, gender, BF, Iron, heme, Hpx, Hpt, *UGT1A1*-genotype.
***HMOX* gene expression:** BMI, *HMOX*-genotype, *HMOX* GT-repeats, *BLVRA* gene expression, gender, BF, UCB, Hpx, Hpt, heme, *UGT1A1*-genotype.
***BLVRA* gene expression:** BMI, *HMOX*-genotype, *HMOX* GT-repeats, *BLVRA* gene expression, gender, BF, UCB, Hpx, Hpt, heme, *UGT1A1*-genotype.
BMI: UCB, *HMOX*-genotype, *HMOX* GT-repeats, *HMOX* gene expression; *BLVRA* gene expression; *UGT1A1*-genotype.
BF: UCB, *HMOX*-genotype, *HMOX* GT-repeats, *HMOX* gene expression; *BLVRA* gene expression; *UGT1A1*-genotype

<u>Supplementary figure S1 a – i.</u> Selected correlations between UCB and other phenotypic characteristics of the BiliHealth study population (all subjects).

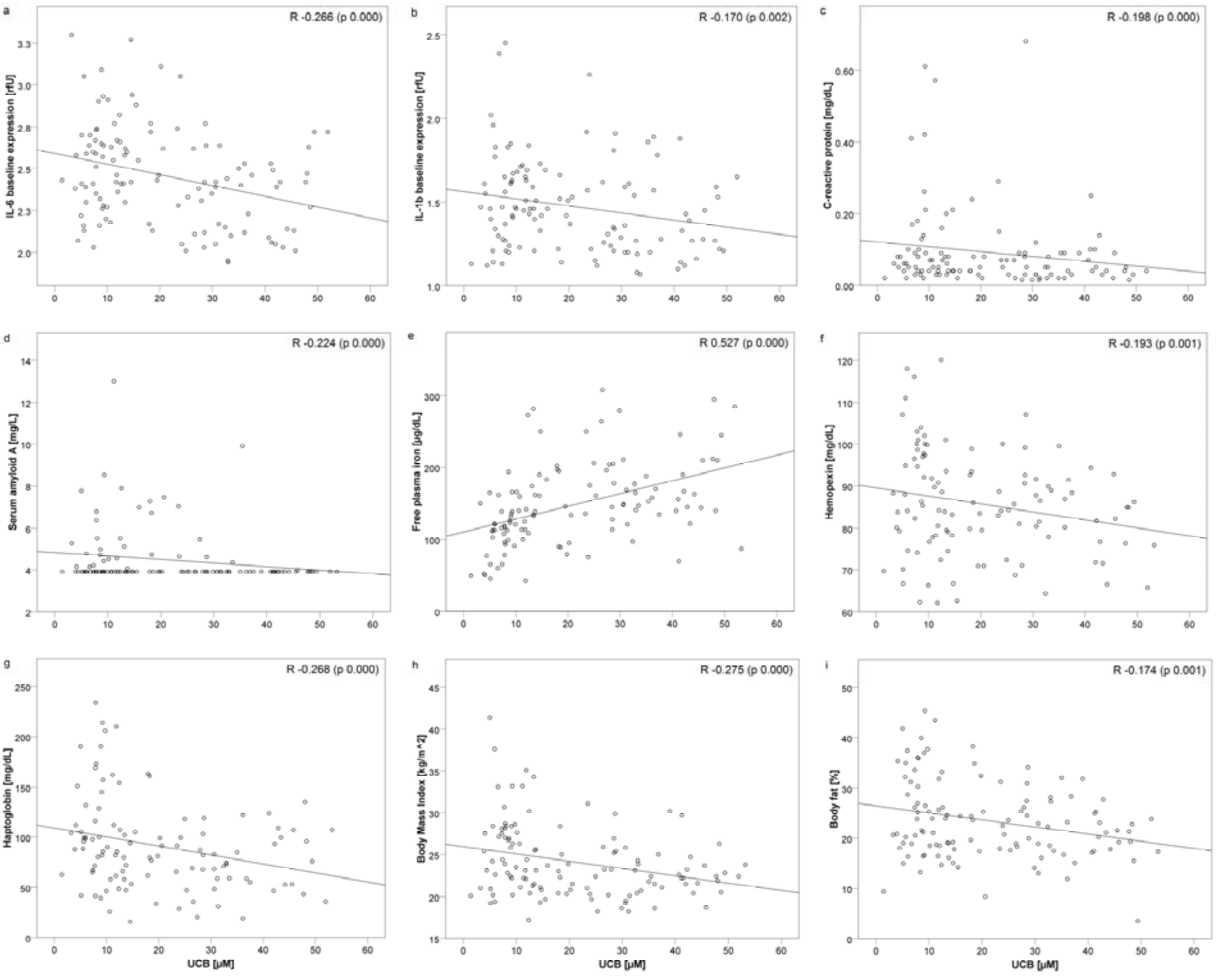

Supplementary Fig. S1 displays correlated measures of an altered heme catabolic pathway in GS. Bivariate correlations between UCB and other phenotypic characteristics of BiliHealth study participants were calculated for all subjects, using the model of Spearman's rho. R coefficients and p-values ($p \leq 0.05$; in brackets) are presented in the figure.

<u>Abbreviations:</u> UCB: unconjugated bilirubin; IL-6/IL-1β: intracellular interleukins 6 and 1β; CRP: C-reactive protein; SAA: serum amyloid A; Hpx: hemopexin; Hpt: haptoglobin; BMI: body mass index; BF: body fat.
